# Supplementary material for: Biphasic response as a mechanism against mutant takeover in tissue homeostasis circuits
Source: Mol Syst Biol. 2017 Jun 26;13(6):933. doi: 10.15252/msb.20177599 (PMC5488663; doi:10.15252/msb.20177599)
Supplement: Supplementary file 1 — Appendix [file MSB-13-933-s001.pdf]

Appendix - Biphasic response as a mechanism against mutant takeover in tissue homeostasis circuits

Table of Contents

APPENDIX - BIPHASIC RESPONSE AS A MECHANISM AGAINST MUTANT TAKEOVER IN TISSUE HOMEOSTASIS CIRCUITS **1**

Appendix Section S1. Modeling the growth rate of cells in tissue homeostasis circuits. *2*

Appendix Section S2. Modeling input delay in feedback homeostasis circuits. *4*

Appendix Section S3. Simulation of glucose dynamics and the induction of a glucokinase mutant. *6*

Appendix Section S4. Derivation of the evolutionary stability of a circuit with biphasic control. *10*

Appendix Section S5. Evolutionary stability of circuits that consist of multiple compartments.. *14*

REFERENCES **17**

Appendix Section S1. Modeling the growth rate of cells in tissue homeostasis circuits.

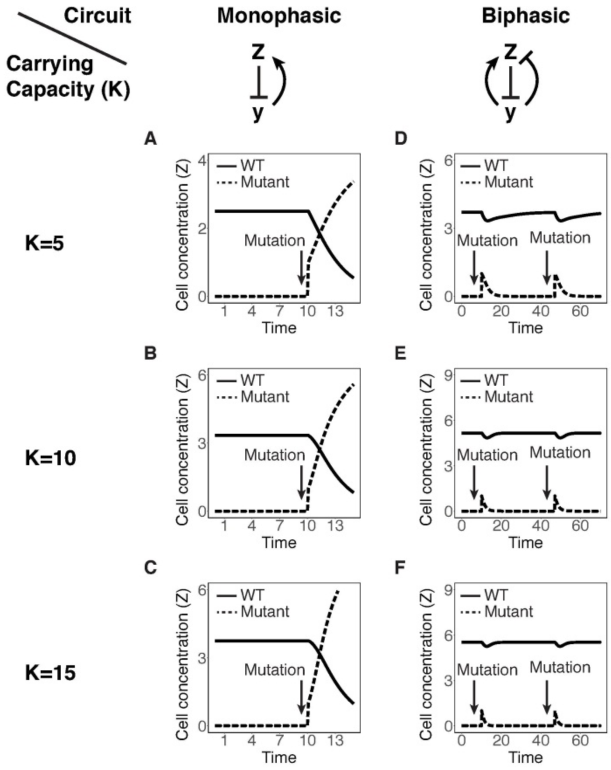

Appendix Figure S1. Adding carrying capacity K to the circuits preserves the conclusions of the study. Simulation of an event where a strong activating mutant arises either in a circuit with monophasic control (A-C) or biphasic control (D-F) with logistic growth with a carrying capacity K. The arrows mark the times when a mutant with a strong activation of the sensing of y arises. As was the case for exponential growth, also under logistic growth the monophasic circuit is susceptible to mutant invasion whereas the biphasic circuit is not.

In this section, we ask whether changing exponential growth to logistic growth in the circuits affects the conclusions. In the main text, we analyzed circuits where cells Z adjust their own growth rate as a function of a signal y, which, in turn, is affected by the size of the tissue. The signal y affects the growth rate of cells by affecting either their proliferation or removal rate, so we can model the dynamics of Z using the following equation:

$$\dot{Z} = Z \cdot (\lambda_+(y) - \lambda_-(y)) \qquad [1]$$

Where λ_+ is the y-dependent proliferation rate of Z and λ_- is the y-dependent removal rate of Z. As discussed the main text, the feedback on Z through y can robustly maintain tissue size, but is susceptible to the invasion of mis-sensing mutants.

The growth rate of Z can be either logistic or exponential. Exponential growth means that the production rate λ_+ does not depend on Z (for example $\lambda_+=y$), and is relevant when the cells are far from carrying capacity. When the cells are closer to carrying capacity, however, a logistic model more appropriately models the dynamics of Z:

$$\dot{Z} = Z \cdot \left(\lambda_+(y) \cdot (1 - \frac{Z}{K}) - \lambda_-(y)\right) \qquad [1]$$

In which proliferation rate drops to zero as cells approach the carrying capacity K.

The conclusions of the manuscript hold both when the growth of the cells is logistic or exponential (Appendix Figure S1): the biphasic circuit is resistant whereas the monophasic circuit is not.

Appendix Section S2. Modeling input delay in feedback homeostasis circuits.

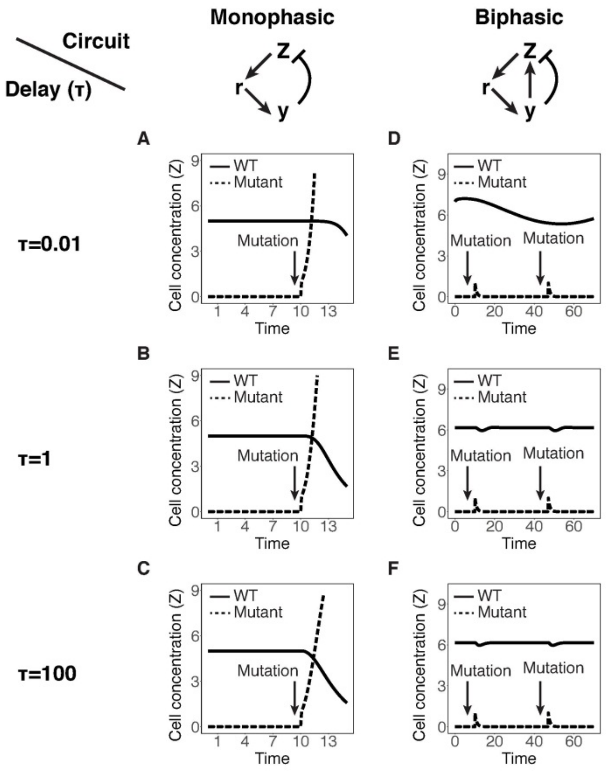

Appendix Figure S2. Simulation of an event where a strong activating mutant arises either in a circuit with monophasic control (A-C) or biphasic control (D-F). The arrows mark the times when a mutant with a strong activation of the sensing of *y* arises. The circuits are similar to the circuits depicted in Fig. 1B and Fig. 1F, except that *Z* acts on *y* with delay modeled by an intermediate variable *r* with delay parameter τ. As was the case without *r*, also here the monophasic circuit is susceptible to mutant invasion whereas the biphasic circuit is not.

In the main text, we analyzed circuits where cells *Z* adjust their own growth rate as a function of a signal *y*, which, in turn, is affected by the size of the tissue. Here, we consider the case where *y* affects *Z* with a delay. Delays occur in endocrine circuits, where the level of the regulated variable (e.g. blood glucose) is controlled with a delay relative to its regulating hormone (insulin).

In the examples of Figure 1 we used the following equations to model the mutant resistance of the circuits in Fig. 1BF:

$$\dot{y} = \mu \cdot (M - (Z + Z_{mut})y) \qquad [1]$$

$$\dot{Z} = Z \cdot (\lambda_{+}(y) - \lambda_{-}(y)) \qquad [2]$$

We tested whether adding a delay to this system affects the resistance of monophasic or biphasic circuits to sensing mutants. To do so, we modify the equations so they include an intermediate variable r with a typical timescale τ:

$$\dot{r} = \tau \cdot (Z + Z_{mut} - r) \qquad [1]$$

$$\dot{y} = \mu \cdot (M - ry) \qquad [2]$$

$$\dot{Z} = Z \cdot (\lambda_{+}(y) - \lambda_{-}(y)) \qquad [3]$$

The parameter τ represents the delay of the system. We tested the effect of 3 different values of τ on the resistance to mutants (Appendix Figure S2) - $\tau=0.01$ (slow), $\tau=1$ (intermediate) and $\tau=100$ (fast). For all these values of τ, an activating mutant invades the monophasic circuit but does not invade the biphasic circuit.

Appendix Section S3. Simulation of glucose dynamics and the induction of a glucokinase mutant.

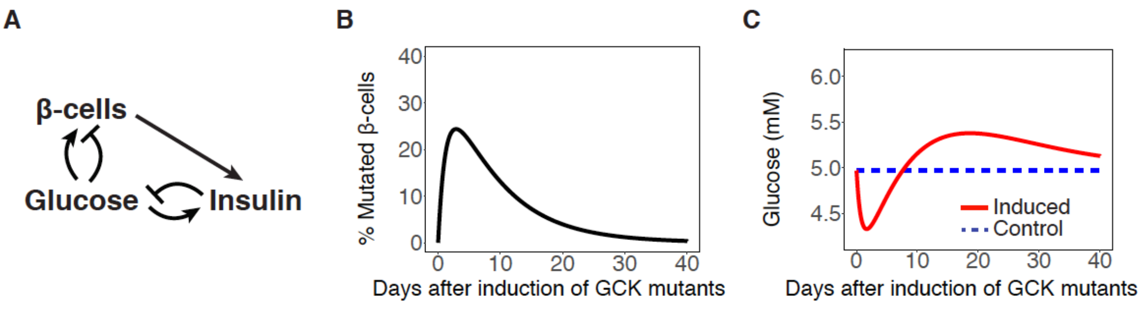

Appendix Figure S3. Simulation of a Tamoxifen-induced conditional knock-in of a 6-fold activating mutant on GCK in beta cells. Dynamics were simulated using explicit equations for insulin and glucose dynamics.

Blood glucose levels are regulated by the hormone insulin which secreted by pancreatic beta cells. The dynamics of glucose as a function of insulin can be described by the following minimal model (Bergman, 1989):

$$\dot{G} = u_0 + u(t) - (C + S_i I) \cdot G \qquad [1]$$

where I is plasma insulin concentration, u_0 is endogenous production of glucose, $u(t)$ is meal intake, C is glucose removal rate at zero insulin and S_i is insulin sensitivity. Secretion of insulin is proportional to beta cell functional mass β and is modeled by the equation:

$$\dot{I} = p\beta \cdot \frac{G^{1.7}}{\alpha^{1.7}+G^{1.7}} - \gamma I \qquad [2]$$

Where $\rho(G)$ is a monotonically increasing function of G, γ is the insulin removal rate and p is the insulin secretion per cell. Last, there is also a slow feedback where glucose controls the dynamics of beta cell proliferation and removal (Karin et al., 2016):

$$\dot{\beta} = \beta(\lambda_+(G) - \lambda_-(G)) = \beta \cdot \lambda(G) \qquad [3]$$

The function $h(G)$ has a stable fixed point at $G = 5mM$. This slow feedback provides the system with robustness to variation in S_i, p since at steady state the dynamics of glucose to any input does not depend on these parameters (e.g. the system shows dynamical compensation (Karin et al., 2016)).

The function *h(G)* also has an unstable fixed point at some $G \gg 5$, which results from glucose-dependant toxicity (glucotoxicity). This unstable fixed point can cause paradoxical beta cell death after an increase in glucose levels, which, in a self-reinforcing manner, further increases glucose levels. This process may underlie type 2 diabetes (De Gaetano et al., 2008; Ha et al., 2016; Karin et al., 2016; Topp et al., 2000). For our simulation, which is intended to represent young mice, we set this unstable fixed point to *G*=13.5mM (Efanova et al., 1998; Maedler et al., 2006). The exact level of the unstable fixed point is not important for our conclusions, since a lower or higher unstable fixed point will work as well (as long as it is significantly smaller than *G*=30mM). We used the following function to model glucose dependent removal of beta cells:

$$\lambda_-(G) = \mu_- \cdot \left(\frac{1}{1+\left(\frac{G}{4}\right)^8} + \frac{1}{1+\left(\frac{15}{G}\right)^6} \right)$$

This death rate is similar to the glucose dependent death curve that is observed by Efanova et al (Efanova et al., 1998). Glucose dependent proliferation rate was modelled as in Karin et al (Karin et al., 2016):

$$\lambda_+(G) = \mu_+ \cdot \frac{1}{1+\left(\frac{8.4}{G}\right)^{1.7}}$$

The values of μ_+,μ_- determine the turnover of beta cell functional mass and were set as:

$$\mu_+ = 0.1 \cdot day^{-1}$$

$$\mu_- = 0.2 \cdot day^{-1}$$

These values correspond to a ~3% turnover of beta cell functional mass per day. All other parameters of the βIG model were set as follows (Karin et al., 2016):

Parameter	Value	Units
u_0	$\frac{1}{30}$	mM min^{-1}

C	10^{-3}	min^{-1}
S_i	$5 \cdot 10^{-4}$	ml μU^{-1} min^{-1}
p	0.03	mg^{-1} μU ml^{-1} min^{-1}
α	8.4	mM
γ	0.3	min^{-1}

A beta-cell mutant with k-fold activation on the sensing of glucose has both a k-fold scaling of insulin secretion ($\rho(G) \to \rho(kG)$) and a k-fold scaling in its response in terms of growth rate ($\lambda(G) \to \lambda(kG)$). Therefore, to simulate the Y214C mutant (that has a 6-fold activation in glucose sensing) we simply replaced the secretion and growth functions accordingly, using $k = 6$. The combined equation for insulin secretion is the following:

$$\dot{I} = p\beta \cdot \frac{G^{1.7}}{\alpha^{1.7} + G^{1.7}} + p\beta_{mut} \cdot \frac{(kG)^{1.7}}{\alpha^{1.7} + (kG)^{1.7}} - \gamma I$$

Finally, in the experiment the Cre-mediated transgene was induced by tamoxifen. We simulated tamoxifen as converting normal beta cells to mutated beta cells:

$$\dot{\beta} = \beta(\lambda_+(G) - \lambda_-(G) - T)$$

$$\dot{\beta}_{mut} = \beta_{mut}(\lambda_+(kG) - \lambda_-(kG))) + \beta T$$

with T representing the concentration of tamoxifen in the blood. The dynamics of tamoxifen were simulated as exponential degradation with a half-life of 16 hours (Robinson et al., 1991) $\dot{T} = \frac{-\log(2)}{16 \cdot 60} T$.

The initial values used for the simulation:

Parameter	Value	Units
T	0.27	day^{-1}
G	4.966667	mM

I	11.42	μU ml^{-1}
β	400	mg
β_{mut}	0	mg

We simulated the dynamics of the system both by (i) assuming a quasi-steady-state for beta cell mass and solving equations [1],[2] to compute glucose levels, and (ii) explicitly modeling the dynamics of glucose and insulin using equations [1], [2], which adds a delay to the circuit. The model was simulated for $t = 40 \cdot 24 \cdot 60$ minutes. The results from (i) are provided in Fig. 1 in the main text and the results from (ii) are provided here as a supplementary figure (Appendix Figure S3). Because beta cell mass changes much slower than glucose, both methods yield highly similar results.

Appendix Section S4. Derivation of the evolutionary stability of a circuit with biphasic control.

Here we derive a formula that approximates the evolutionary stability of a circuit with biphasic control. First, we provide a definition for the evolutionary stability of a circuit.

Definition 1.1. The *strategy* S of a cell is defined as its (daily) death and proliferation probabilities for all inputs c: $S=\{d(y),p(y)\}$. A strategy $S'=\{d'(y),p'(y)\}$ is denoted as an alternative strategy to S if there are inputs y for which either $p'(y) \neq p(y), d'(y) \neq d(y)$. The strategy that is adopted by a population of cells, as well as the output response of these cells to the input y, determines the function of the homeostatic circuit.

Definition 1.2. The *evolutionary stability* of a strategy S is defined as the probability that given that the entire population of cells adopts S, it will not be invaded by an alternative strategy S' by time t.

If the entire population adopts a strategy S then for an alternative strategy S' to invade the circuit, it must first arise via mutation. The probability that S' will arise via mutation from a cell with a strategy S is the probability of a transition $S \rightarrow S'$, which we denote it as $\mu_S(S')$. After a mutant arises, it must then invade the population. We denote the probability that such a mutant will invade the population as $\rho_S(S')$.

For a circuit of N interacting cells with a turnover rate τ^{-1}, the probability that no invading alternative strategy will arise by time t is:

$$\zeta_S(t) = \left(\prod_{S'} \left(1 - \mu_S(S')\rho_S(S')\right)\right)^{N\tau^{-1}t} \quad [1]$$

We estimate the invasion probability $\rho_S(S')$ by modeling the evolutionary dynamics of the circuit using a Moran-like stochastic process with variable death and proliferation probabilities.

Definition 1.2. Consider a population of cells with strategies $\{S_1, \ldots, S_N\}$ with corresponding death and proliferation probabilities $S_i=\{d_i,p_i\}$. The *Extended Moran Process (EMP)* is defined as follows: in each round, one strategy replicates and one strategy is eliminated. The probability for elimination for strategy S_i is $\frac{d_i}{\sum_{j=0}^{N} d_j}$ and the probability for replication is $\frac{p_i}{\sum_{j=0}^{N} p_j}$. The relative fitness of a strategy is defined as $r = \frac{p_i}{d_i}$.

Lemma 1.1. The probability that a strategy S_i will take over the population in the Extended Moran Process is: $\rho_{S_i} = \frac{1-\frac{1}{r}}{1-\frac{1}{r^N}}$.

Proof. The proof is the same as the proof for the fixation probability of the standard Moran Process (Nowak, 2006), by simply setting the death probability as non-constant.

Theorem 1.1. The probability that an alternative strategy $S'=\{d'(y),p'(y)\}$ invades a homeostatic circuit is:

$$\rho_S(S') \approx \frac{1-\frac{1}{r}}{1-\frac{1}{r^N}} \quad [2]$$

where $r = \frac{p'(y_{ST})}{d'(y_{ST})}$ with $y=y_{ST}$ being the homeostatic set-point, and N being the number of cells in the original population at steady-state. For a $N>10$ and $r>1$ this is approximately $\rho_S(S'|r > 1) \approx 1 - \frac{1}{r}$, while for $r<1$ the invasion probability approaches zero: $\rho_S(S'|r < 1) \approx 0$.

Proof. This result follows directly from *Lemma 1.1* when we model the evolutionary dynamics of the population using EMP. Note that the assumptions of EMP are not met precisely – if cells with S' proliferate more rapidly than cells with S die, then the population size exceeds N and $y \neq y_{ST}$ before S' invades the circuit. Nevertheless, for a mutant with a fitness advantage, the highest probability of elimination occurs when its frequency is

relatively small, and thus when the overall number of cells is approximately *N* and the $y \approx y_{ST}$.

Next we analyze the transition probability $\mu_S(S')$ for some alternative strategy S'. We assume that the transition $S \to S'$ results from random mutations in enzymes. Consider such an enzyme with an output that is described by a Hill equation: $f(x) = v_m \frac{1}{1+(Kx^{-1})^n}$. A mutation that changes $K \to K'$ results in a scaled output response:

$$f'(x) = v_{max}\frac{1}{1+(K'x^{-1})^n} = v_{max}\frac{1}{1+(K\cdot(x^{-1}K^{-1}K'))^n} = f(x^{-1}K^{-1}K')$$

We define $\chi = K^{-1}K'$. In a circuit with biphasic control where the enzyme is upstream both death and proliferation this mutation will result in an alternative strategy *S'={d(χy),p(χy)}*. Other mutations that change v_{max} or n may have an effect on input sensing that is not necessarily scaling (the effect depends on the structure of the signaling network), but for the simplicity of the analysis we approximate the effect of every mutation as if it scales the input.

Thus, every mutation that affects sensing corresponds to some scaling value χ. The scaling χ can range from $\chi = 0$ (locked off) to $\chi = \infty$ (locked on) with $\chi = 1$ being a neutral mutation with respect to sensing. We define the probability density function $P_S(\chi)$ over $\chi \in [0,\infty]$ to approximate the probability that a mutant with a scaling χ will arise by mutation from a population with strategy S. The measure $\mu_S(\chi)$ is defined to be equal to $P_S(\chi)$ for all $\chi \neq 1$ and $\mu_S(1) = 0$. In addition, for every scaling χ we can infer the invasion probability using [Theorem 1.1]:

$$r(\chi) = \frac{p(\chi y_{ST})}{d(\chi y_{ST})} \quad [3]$$

The evolutionary stability of the circuit can be inferred by integrating over all possible values of χ:

$$\zeta_S(t) = e^{N\tau^{-1}t\int_0^\infty \log(1-\mu_S(\chi)\rho_S(\chi))d\chi} \quad [4]$$

To further simplify the analysis we use a zero-order approximation of $\mu_S(\chi) = \mu_0$ around $\chi = 1$, which is the relevant range where sensing mutants may have a fitness advantage over wild-type cells. The value of μ_0 depends both on DNA replication fidelity and on the number of mutations that affect χ, which may be, for instance, the number of mutations that affect the kinetics or expression level of a rate-limiting enzyme.

Let us now consider a circuit with biphasic control, an unstable fixed point y_{UST} and a stable fixed point y_{ST}. The input range where proliferation exceeds death is $y_{ST} \leq y \leq y_{UST}$ and we assume a step change in that range so $\frac{p(y)}{d(y)} = \nu$ in that range. The population size is $N > 10$ and we denote $\delta = \frac{y_{UST}}{y_{ST}} - 1$. Thus, only for $1 < \chi < 1 + \delta$ is the invasion probability $\rho_S(\chi)$ non-negligible and is equal to $\rho_S(\chi) = 1 - \frac{1}{\nu}$. The evolutionary stability of this circuit is thus:

$$\zeta_S(t) = e^{N\tau^{-1} t \int_1^{1+\delta} \log\left(1 - \mu_0\left(1 - \frac{1}{\nu}\right)\right) d\chi} \approx e^{N\tau^{-1} t \int_1^{1+\delta} \mu_0\left(1 - \frac{1}{\nu}\right) d\chi}$$

$$= e^{N\tau^{-1} t \delta \mu_0\left(1 - \frac{1}{\nu}\right)}$$

Appendix Section S5. Evolutionary stability of circuits that consist of multiple compartments.

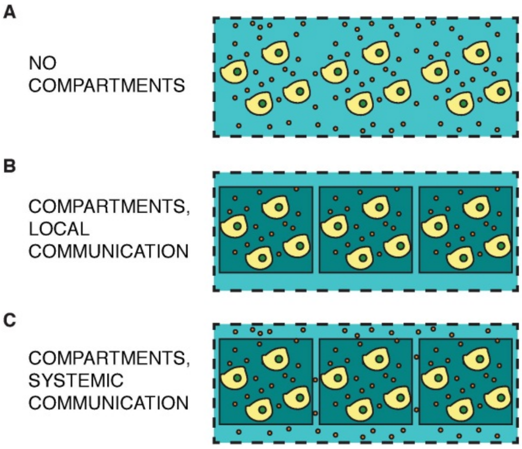

Appendix Figure S4. Architectures of compartmentalizations for intercellular communication.

Tissues of multicellular organisms are often subdivided into compartments such as intestinal crypts or pancreatic islets (Jo et al., 2007; Michor et al., 2003; Mintz, 1971). The number of cells in each compartment is limited and cannot exceed a certain size, and thus they confine invading mutants. We now extend our analysis to the case where the cells in a circuit are subdivided into many compartments (Appendix Figure S4). In addition to the non-compartmentalized case (Appendix Figure S4A), there are two possible scenarios: either the cellular communication is local to each compartment (Appendix Figure S4B, e.g. paracrine signaling) or the communication is systemic, between compartments (Appendix Figure S4C, e.g. the control of metabolites by endocrine tissues).

Local communication: If each compartment is of similar size then each compartment has the same evolutionary stability ζ. This value ζ is the same as the expected fraction of compartments that have an invading mutant by time t.

Systemic communication: Consider now the case where the communication occurs across the entire population and the circuit has only one non-trivial stable fixed-point. If a mutant invades a compartment then the homeostatic set point for all the cells in that compartment is different from that of the rest of the population. Recall that in the non-compartmentalized

case the system can be at steady state only when all the cells of the population have the same strategy (that is, the same scaling χ). This is not the case when the population is subdivided into compartments. The reason for this is that while the cells in the invading compartment may have a positive growth rate when the system is at its original homeostatic set-point, they cannot grow beyond the limits of their compartment unless they acquire additional mutations. Thus, if the fraction of invaded compartments is small then the original homeostatic set-point is still maintained and the fraction of invaded compartments is the same as the evolutionary stability of each compartment: ζ.

What is the optimal compartment size for a population of N cells? As is the case in the somatic evolution of cancer (Michor et al., 2003) there is a tradeoff between large and small compartments. Large compartments are more robust against random drift while small compartments are more robust against mutants with a fitness advantage. To illustrate why this is the case we consider two extremes: very large and very small compartments. If the compartments are very large then, in case an invading mutant arises, it takes over a large part of the population and has a larger effect on circuit function. Very small compartments, on the other hand, are more susceptible to being taken over by mutants that are neutral or have a fitness disadvantage (Michor et al., 2003).

References

Bergman, R.N. (1989). Lilly lecture 1989. Toward physiological understanding of glucose tolerance. Minimal-model approach. Diabetes *38*, 1512–1527.

De Gaetano, A., Hardy, T., Beck, B., Abu-Raddad, E., Palumbo, P., Bue-Valleskey, J., and Porksen, N. (2008). Mathematical models of diabetes progression. AJP Endocrinol. Metab. *295*, E1462–E1479.

Efanova, I.B., Zaitsev, S.V., Zhivotovsky, B., Köhler, M., Efendić, S., Orrenius, S., and Berggren, P.O. (1998). Glucose and tolbutamide induce apoptosis in pancreatic beta-cells. A process dependent on intracellular Ca2+ concentration. J. Biol. Chem. *273*, 33501–33507.

Ha, J., Satin, L.S., and Sherman, A.S. (2016). A Mathematical Model of the Pathogenesis, Prevention, and Reversal of Type 2 Diabetes. Endocrinology *157*, 624–635.

Karin, O., Swisa, A., Glaser, B., Dor, Y., and Alon, U. (2016). Dynamical compensation in physiological circuits. Mol. Syst. Biol. *12*, 886.

Maedler, K., Schumann, D.M., Schulthess, F., Oberholzer, J., Bosco, D., Berney, T., and Donath, M.Y. (2006). Aging correlates with decreased beta-cell proliferative capacity and enhanced sensitivity to apoptosis: a potential role for Fas and pancreatic duodenal homeobox-1. Diabetes *55*, 2455–2462.

Nowak, M.A. (2006). Evolutionary dynamics: exploring the equations of life (Cambridge, Mass: Belknap Press of Harvard University Press).

Robinson, S.P., Langan-Fahey, S.M., Johnson, D.A., and Jordan, V.C. (1991). Metabolites, pharmacodynamics, and pharmacokinetics of tamoxifen in rats and mice compared to the breast cancer patient. Drug Metab. Dispos. Biol. Fate Chem. *19*, 36–43.

Topp, B., Promislow, K., deVries, G., Miura, R.M., and Finegood, D.T. (2000). A model of beta-cell mass, insulin, and glucose kinetics: pathways to diabetes. J. Theor. Biol. *206*, 605–619.
