# Supplementary material for: Biphasic response as a mechanism against mutant takeover in tissue homeostasis circuits
Source: Mol Syst Biol. 2017 Jun 26;13(6):933. doi: 10.15252/msb.20177599 (PMC5488663; doi:10.15252/msb.20177599)
Supplement: Supplementary file 2 — Expanded View Figures PDF [file MSB-13-933-s002.pdf]

Expanded View Figures

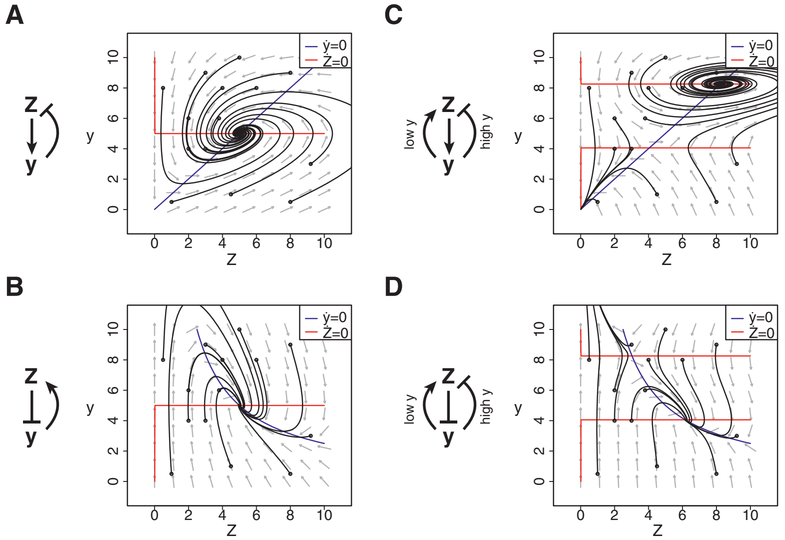

Figure EV1. Phase plots of alternative tissue feedback circuits.

- A A monophasic feedback circuit in which cells *Z* generate an input *y* that inhibits their growth rate. Trajectories from different initial concentrations of *Z*,*y* reach steady state at $Z = Z_{ST}$, $y = y_{ST}$.
- B A monophasic feedback circuit in which cells *Z* inhibit *y* which increases their growth rate. Trajectories from different initial concentrations of *Z*,*y* reach steady state at $Z = Z_{ST}$, $y = y_{ST}$.
- C A biphasic feedback circuit where *Z* generates a signal *y*, which, in turn, decreases the growth rate of *Z* at high concentrations and increases the growth rate of *Z* at low concentrations. Trajectories from different initial concentrations of *Z*,*y* either reach steady state at $Z = Z_{ST}$, $y = y_{ST}$, or, at low initial *Z* or high initial y, result in population collapse $Z \rightarrow 0$
- D A biphasic feedback circuit where cells *Z* inhibit *y*, which, in turn, decreases the growth rate of *Z* at high concentrations and increases the growth rate of *Z* at low concentrations. Trajectories from different initial concentrations of *Z*,*y* either reach steady state at $Z = Z_{ST}$, $y = y_{ST}$, or, at low initial *Z* or low initial y, result in population collapse $Z \rightarrow 0$.

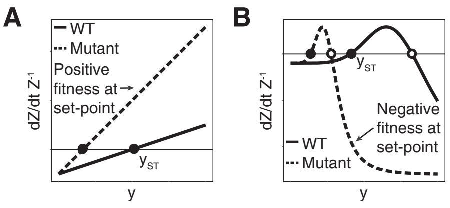

Figure EV2. Sensing mutations modulate the effect of input on growth rate.

- A A monophasic feedback circuit in which cells *Z* inhibit *y* which increases their growth rate (solid line). A threefold activating mutation causes a shift in the response curve (dashed line), so the mutant mis-senses the level of *y* to a higher level where the mutant has a positive growth rate.
- B A biphasic feedback circuit where cells *Z* inhibit *y*, which, in turn, decreases the growth rate of *Z* at high concentrations and increases the growth rate of *Z* at low concentrations (solid line). A threefold activating mutation causes a shift in the response curve (dashed line), so the mutant mis-senses the level of *y* to a higher level where the mutant has a negative growth rate.

© 2017 The Authors

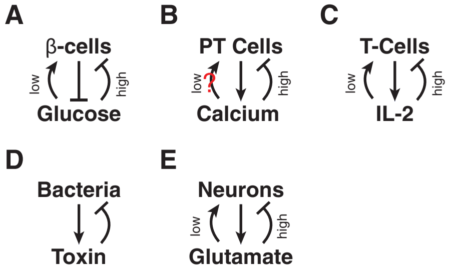

Figure EV3. Schematic diagrams for tissue homeostasis circuits.

A Plasma glucose inhibits beta cell death at low concentrations and is toxic for beta cells at high concentrations. Beta cells, in turn, secrete insulin which reduces the level of plasma glucose.

B Plasma calcium inhibits parathyroid cell proliferation at high concentrations and may potentially stimulate parathyroid cell growth at low concentrations. Parathyroid cells, in turn, secrete PTH which increases the level of plasma calcium.

C T cells secrete IL-2, which affects both T-cell death and proliferation such that growth is negative at high and low concentrations of IL-2.

D Secrete-and-sense circuit engineered by You *et al* (2004), where a death promoter in bacteria is under the control of a molecule secreted by the bacteria.

E Neurons secrete glutamate. Glutamate is toxic to neurons at high concentrations and promotes neuronal survival at low concentrations.

 © 2017 The Authors
